# Evaluation of dose distributions in gamma chamber using glass plate detector

**DOI:** 10.4103/0971-6203.41277

**Published:** 2008

**Authors:** Pradeep Narayan, S. G. Vaijapurkar, P. K. Bhatnagar

**Affiliations:** Defence Laboratory, Ratanada Palace, Jodhpur, Rajasthan, India

**Keywords:** Dose distribution, flat glass plate, gamma chamber, high dose measurement, optical densitometry

## Abstract

A commercial glass plate of thickness 1.75 mm has been utilized for evaluation of dose distributions inside the irradiation volume of gamma chamber using optical densitometry technique. The glass plate showed linear response in the dose range 0.10 Kilo Gray (kGy) to 10 kGy of cobalt-60 gamma radiation with optical sensitivity 0.04 Optical Density (OD) /kGy. The change in the optical density at each identified spatial dose matrix on the glass plate in relation to the position in the irradiation volume has been presented as dose distributions inside the gamma chamber. The optical density changes have been graphically plotted in the form of surface diagram of color washes for different percentage dose rate levels as isodose distributions in gamma chamber. The variation in dose distribution inside the gamma chamber unit, GC 900, BRIT India make, using this technique has been observed within ± 15%. This technique can be used for routine quality assurances and dose distribution validation of any gamma chamber during commissioning and source replacement. The application of commercial glass plate for dose mapping in gamma chambers has been found very promising due to its wider dose linearity, quick measurement, and lesser expertise requirement in application of the technique.

## Introduction

The quality of radiation-processed materials (blood, animal, medical and R and D) depends on the accuracy of the radiation dose delivered to the product. For commercial applications, generally larger volumes are irradiated and thus dose is not as uniform as for small volumes used for research applications. The dose variations in the gamma irradiators/chambers are unavoidable and depend on the type of source geometry of the irradiation volume/chamber. The main objective of dose mapping is to determine the maximum and minimum dose in the container and irradiation volume. Chemical (Fricke and Ceric-Cerous), ESR (Electron Spin Resonance), Film, and TL (thermoluminescence) are the main processes on which high dose (kGy range) dosimetry is based. Applications of general-purpose glasses have been reported for high dose measurement using TL and OD (optical densitometry) techniques in the recent past[3,4] The dose distributions in the irradiation volume of gamma chamber are not perfectly homogeneous, and variations have been reported within ±35% in some of the irradiators.[6] This variation in dose rate within the irradiation volume is due to typical source-irradiation chamber geometry. Source pencils are arranged in an annular ring which surrounds the cylindrical irradiation chamber and hence the product, during irradiation. If dose in-homogeneity within the chamber is not under one's control, at least it should be known for dosimetry correction and uncertainty evaluation in the irradiated samples.

Selection of a suitable dosimetry system depends on several considerations, such as; dose range of interest, the precision and ease of measurement, the expertise available, environmental factors that can be important at the location of use, cost, and uncertainty that is consistent with the process. The primary purpose of performing dose mapping is to verify that the dose variability in the irradiated sample is acceptable for the application on hand. This should be done before useful irradiation is carried out. If the distribution is wider than acceptable, it points out the need for modifying the irradiation procedure or the container size/shape. The detailed dosimetry is generally carried out by carefully placing several dosimeters inside the irradiated volume.

Some of the authors have used chemical dosimeters for dose distribution studies of gamma cell. The doses at circumference of exposure chamber have been found to be more by 15% to 22% compared to those at the center, either at ground plane or midplane of irradiation chamber.[5] The central dose at midplane of gamma chamber (GC 5000, BRIT, India) has been 20% more compared to that at the ground plane of the chamber.[5] The ISP DOSE-MAP dosimetry system for blood irradiators is based on an instant-imaging film medium that darkens in response to ionizing radiation. The calibrated optical densitometry films are capable of measuring the absorbed dose in a blood irradiator canister to a level of accuracy of the order of ±5%. The DOSE-MAP cassette is placed into the irradiation canister, and the canister is filled with water during exposure in gamma chamber. The cassette/water “phantom” is then subjected to a standard irradiation cycle. This dosimetry system provides validation of irradiator performance for the 25 Gy central dose target with 15 Gy minimum dose limit. In commercial blood irradiators, the dose delivered can vary from the center of the container to the periphery by up to 35%; and along the central axis, by up to 30%.[2] Chemical dosimeters, such as alanine/glutamine (spectrophotometric readout), Fricke, and FBX, covering a broad range provide useful tools for dose mapping.[1] The evaluation of variation of dose distribution in biological, blood, and R and D materials is very important as this can drastically affect the result and the quality of radiation-processed product.

Authors of this paper have used general-purpose plain glass plate for evaluation of dose distribution in gamma chamber (GC 900), BRIT India, using optical densitometry technique for the results presented in this paper. This glass plate has also been subjected to linearity study and fading under different environmental conditions before using it for dose distribution study.

## Experimental procedure

A flat commercial glass plate of thickness 1.75 mm with trace elements impurities concentration — Cu-0.01%; Mn-0.02%; Fe-0.68%; Zn-5.31%; Mg-2.60%; Na-14.65%; K-12.56%; and Ca-2.10% — has been selected for this study. The glass plate was cut into square pieces of size 20 mm × 20 mm and irradiated in cobalt-60–based gamma chamber with central dose rate 125 Gy/h for different doses varying from 100 Gy to 85 kGy. Five glass detectors were used for each dose measurement and each irradiated detector read at five locations (center and four corners). In this way, 25 readings were taken at each dose measurement. After exposure, the samples were covered with black, thick paper to restrict ambient light exposure to the glass samples. The exposed samples were read in an optical densitometer (model: Speedmaster SM-10TI) with resolution ±0.01 OD. The average OD reading and uncertainty in measurement at 95% confidence level have been calculated for response of the glass detector at different dose levels. A graph was plotted between dose and changes in optical density. The dose range of the linear portion of the response curve was selected for further studies on fading and dose distribution. Since 5 kGy of cobalt-60 gamma radiation had been observed to lie in the mid of the linear response curve of the glass, this dose level was chosen for further exposure of the sample for dose distribution study. Ten glass detectors were irradiated with 5 kGy of gamma radiation dose and subjected to fading study under different environmental conditions. The samples were stored in different environmental conditions such as under dark and room temperature, room light and room temperature, room light and 50°C, room light and 70°C, and solar exposure and read after 5 h of irradiation. The fading contribution due to various environmental factors is evaluated and reported.

For dose distribution evaluation study, the glass plate was cut identical to the size of central axial plane (140 mm × 90 mm) of the gamma chamber. The plate was then vertically positioned along the central axis of gamma chamber with the help of flat thermo-coal base, properly immobilized, and exposed with 5 kGy of gamma dose. After completion of irradiation, the glass plate was removed from the chamber and covered with black, thick paper to avoid visible light exposure. The surface of the exposed glass plate was marked with fine marker pen to identify all its matrix positions in relation to the position in gamma chamber with matrix size (5 mm × 5 mm). Each identified and marked matrix position was read in the optical densitometer with aperture of 2 mm diameter and the change in the density at each position noted. A surface diagram using MS Excel-based program has been plotted between each matrix position in relation to gamma chamber spatial positions. The relative changes in optical density of glass plate at each matrix position with respect to chamber central optical density have been calculated in terms of percentage. The variations in the percentage OD with respect to central OD have been presented as the color washes. The variation in the relative optical density gives the measure of the variation in dose rate within the gamma chamber irradiation volume. The same procedure has been repeated for different chamber axial planes at different angles to get dose distribution inside the irradiation volume of the chamber.

## Result and Discussion

### Optical response of gamma-exposed glass plate

**Figure 1:**
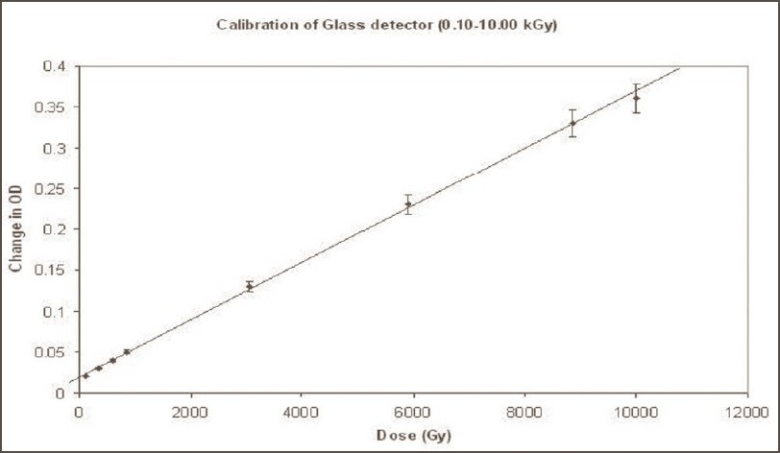
Optical density changes of the glass plate exposed with cobalt-60 gamma radiation (100 Gy-10 kGy)

The glass plate studied under this investigation showed linear response in the dose range 100 Gy to 10 kGy, with OD sensitivity 0.04 OD/kGy and measurement error ±5% at 95% confidence level; sub-linear response in the dose range 10 kGy to 50 kGy; and saturation beyond 50 kGy. The net change in the absorbance of the glass detectors at each dose point is given in Table 1. The calibration graph of glass detector in the dose range 0.10 kGy to 10.00 kGy is shown in Figure 1. The central dose (5 kGy in this case) as per linear OD response of glass detectors against gamma rays, should be selected for evaluating both under-dose and overdose rate positions in comparison to central dose rate of gamma chamber.

**Table 1:** Calibration of glass detector (0.10 kGy-10.00 kGy) against cobalt-60 gamma radiation

*Dose (Gy)*	*Net change in absorbance (OD)*	*Variation in absorbance at 95% confidence level (CL) within 20×20 mm^2^ area of the detector*	*% Variation in absorbance at 95% CL within 20×20 mm^2^ area of the detector*
100	0.02	0.00	0.00
350	0.03	0.00	0.00
600	0.04	0.00	0.00
850	0.05	0.00	0.00
3050	0.13	0.00	0.00
5900	0.23	0.01	4.35
8850	0.33	0.01	3.03
10000	0.36	0.01	2.78

## Optical fading

The irradiated (5 kGy) glass samples showed 14% fading of absorbance in the first 24 h of exposure under normal environmental conditions. However, fading of the exposed glass in 5 h was observed to be 3%, 4%, 29%, 58%, and 60% under dark and room temperature, room light and room temperature, room light and 50°C, room light and 70°C, and solar exposure respectively. Necessary corrections can be applied accordingly in case the exposed samples encounter such environmental conditions. The fading issues can be avoided by storing the samples under identical environmental conditions and reading at identical interval of time after irradiation.

## Dose distribution in irradiation volume

**Figure 2:**
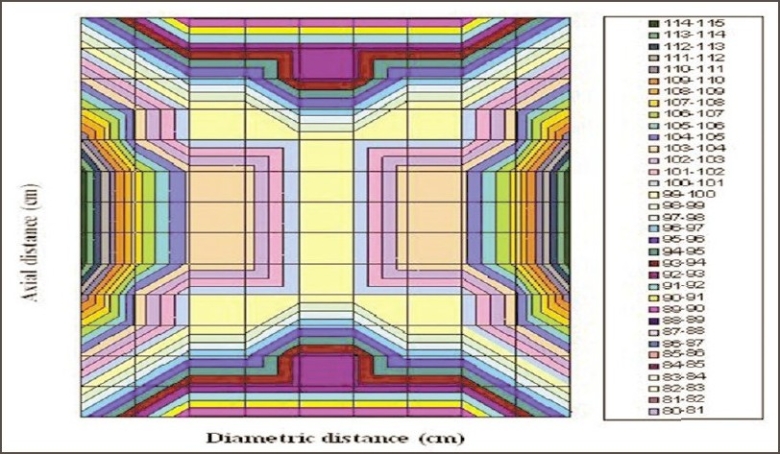
Dose distribution in the irradiation volume of gamma chamber (GC 900), BRIT India, using glass plate exposed with 5 kGy of cobalt-60 gamma radiation

**Figure 3:**
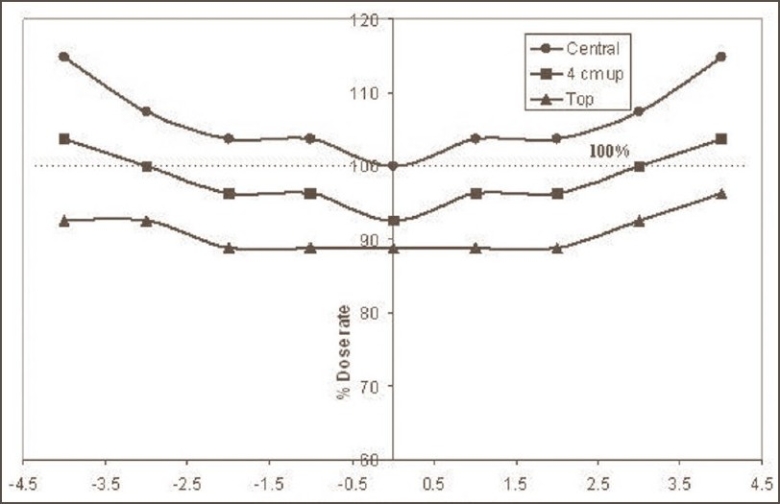
Variation of dose rate along the diameter of cylindrical irradiation volume of gamma chamber (GC 900), BRIT India

**Figure 4:**
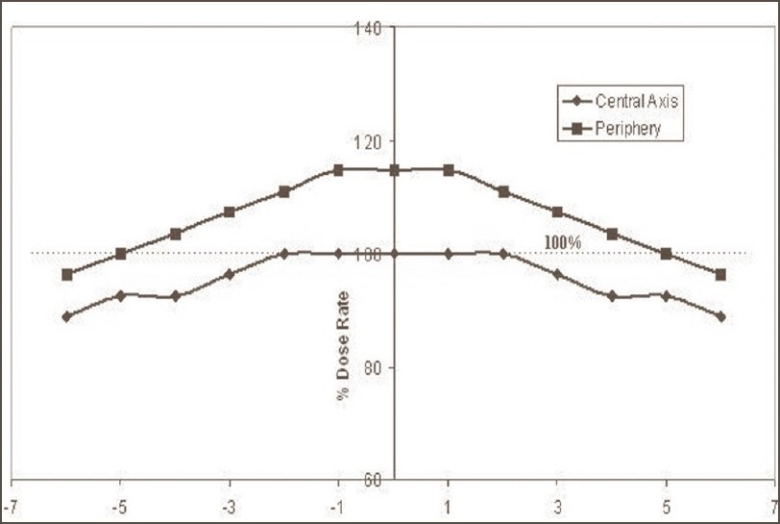
Variation of dose rate along the central axis of cylindrical irradiation volume of gamma chamber (GC 900), BRIT India

The dose distribution inside the irradiation volume of the gamma chamber is shown in Figure 2. The variation in the dose rate along the diametrical position of the cylindrical chamber was found to be within ±15% in comparison to the central dose rate; however, this variation along the direction of central axis was within ±10%. This variation is a very important factor in case of animal experiments and blood irradiation in stationary mode. The dose rate within the concentric cylindrical volume [50 mm (L) × 15 mm (Φ)] was almost homogeneous within ±1%; however, as the size of the concentric cylindrical irradiation volume increases, variations in the dose rate also increase. The dose rate variations for concentric cylindrical irradiation volumes [70 mm (L) × 60 mm (Φ)] and [90 mm (L) × 70 mm (Φ)] were ±4% and ±6% respectively with respect to central dose rate. The central cylindrical volume with diameter 5 cm and length 8.0 cm was found to receive radiation doses within ±5% and can be acceptable in most of R and D material irradiation. Figures 3 and 4 show the variation in dose rate within the irradiation volume of gamma chamber along diametric and axial planes respectively and can help in judging the absorbed dose in bigger size samples.

## Conclusion

The glass studied in this investigation has wide linear OD response against gamma radiation, typically 0.10 kGy to 10 kGy. However, individual glass material requires calibration and study of properties as done here. Any commercial flat glass plate with moderate OD sensitivity against gamma radiation can be used as a routine radiation field analyzer for evaluation of dose distribution in gamma chamber. This technique is very simple and requires only one instrument, viz., optical densitometer. Glasses do not require any buildup or scattering media as in case of film densitometry, in which water media is required to fill around the film cassette. Only one glass plate detector covering the whole axial plane of the chamber is capable of giving the information about dose distribution inside the whole chamber. Whereas chemical and thermoluminescent (TL) dosimetry systems for dose mapping require many detectors of definite size and geometry to be put inside the chamber, which adds up more uncertainty to the dose distribution analysis.

The graphical dose distributions as shown in Figure 2 can be used as a tool for estimating the dose rate at any point inside the chamber. Precise chemical dosimetry or any secondary standard dosimetry at only one point inside the irradiation volume is sufficient to have dose rate information at all points inside the total irradiation volume with the aid of glass plate dose distribution. The glasses are chemically inert and have very little environmental effects in comparison to other high dose dosimetry systems. Any flat transparent glass with proper thickness, which can be accommodated in the optical densitometer, can be used to estimate dose profile in the gamma chamber. High-resolution optical densitometer with scanning facility may give better-resolved dose profile of gamma chamber.
